# Spinning faster: protein NMR at MAS frequencies up to 126 kHz

**DOI:** 10.1007/s10858-018-0219-9

**Published:** 2019-01-24

**Authors:** Susanne Penzel, Andres Oss, Mai-Liis Org, Ago Samoson, Anja Böckmann, Matthias Ernst, Beat H. Meier

**Affiliations:** 1 Physical Chemistry, ETH Zürich, Vladimir-Prelog-Weg 2, 8093 Zurich, Switzerland; 2 NMR Instituut, Tartu Teaduspark, Tehnomeedikum, Tallinn University of Technology, Akadeemia tee 15a, 19086 Tallinn, Estonia; 3 Institut de Biologie et Chimie des Protéines, UMR 5086 CNRS/Université de Lyon 1, Labex ECOFECT, 7, Passage du Vercors, 69367 Lyon, France

**Keywords:** Fast MAS, Proton detection, Solid-state NMR

## Abstract

We report linewidth and proton *T*_1_, *T*_1*ρ*_ and *T*_2_′ relaxation data of the model protein ubiquitin acquired at MAS frequencies up to 126 kHz. We find a predominantly linear improvement in linewidths and coherence decay times of protons with increasing spinning frequency in the range from 93 to 126 kHz. We further attempt to gain insight into the different contributions to the linewidth at fast MAS using site-specific analysis of proton relaxation parameters and present bulk relaxation times as a function of the MAS frequency. For microcrystalline fully-protonated ubiquitin, inhomogeneous contributions are only a minor part of the proton linewidth, and at 126 kHz MAS coherent effects are still dominating. We furthermore present site-specific proton relaxation rate constants during a spinlock at 126 kHz MAS, as well as MAS-dependent bulk *T*_1ρ_ (^1^H^N^).

**Electronic supplementary material:**

The online version of this article (10.1007/s10858-018-0219-9) contains supplementary material, which is available to authorized users.

## Introduction

Magic-angle spinning (Andrew et al. 1958; Lowe 1959) is a requirement to obtain highly resolved protein spectra with good sensitivity in solid-state NMR that can provide the detailed information needed for structure determination or dynamical analysis. The definition of what is considered fast MAS, however, is constantly changing. Significant progress has been made since 2003 when 30 kHz MAS was initially used for protein spectroscopy (Ernst et al. 2003). At this frequency, carbon detection with high-power decoupling was most efficient for biomolecular NMR. This type of experiments is, however, often hampered by long measurement times, and the demand for relatively large amounts of protein (several tens of milligrams). Compared to carbon detection, proton detection—the standard in solution-state NMR—offers many advantages also in solid-state NMR. The higher gyromagnetic ratio of ^1^H leads to a factor of eight in the signal integral. However, the dense network of strong homonuclear ^1^H–^1^H dipolar interactions leads to line broadening and concomitant low sensitivity at spinning frequencies in use at this time. With a spinning frequency around 50 kHz, not only carbon-detected experiments with low-power decoupling (Ernst et al. 2004) and using exclusively low-power irradiation (Lange et al. 2009; Vijayan et al. 2009) became feasible, but also proton-detected protein NMR in deuterated and partially back-exchanged proteins started to become competitive (Huber et al. 2011; Barbet-Massin et al. 2014a, b; Linser et al. 2008, 2011a, b; Reif and Griffin 2003; Chevelkov et al. 2006; Agarwal et al. 2006). A further significant improvement was obtained with spinning frequencies in the range of 90–111 kHz where the proton linewidths for deuterated and fully back-protonated proteins was narrowed to around 20 Hz for the narrowest lines in proteins (Agarwal et al. 2014). At the same time, the proton resolution for fully protonated proteins is reduced to about 120 Hz, and structural information can be extracted (Andreas et al. 2016; Stanek et al. 2016). Recently spinning at 140 kHz has been described (Lin et al. 2018).

Here, we present protein spectra at MAS frequencies up to 126 kHz. While, the faster (spinning) the better has been predicted (Böckmann et al. 2015), no systematic MAS frequency-dependent data has yet been available in the spinning regime above 111 kHz. We explore this perspective here and compare, using ubiquitin as a model, experimental proton linewidths and echo-detected coherence-decay times *T*_2_′ of the amide protons in fully protonated ubiquitin at 93 and 126 kHz MAS, as well as MAS-dependent bulk relaxation parameters. The predicted improvement expected for MAS frequencies above the widely used 111 kHz is experimentally demonstrated.

## Theoretical background

To quantify the linewidth of a peak in the spectrum Δ^tot^—measured in frequency units as full width at the half maximum (FWHM)—we distinguish three different contributions. Note that the relationship between the relaxation time $T_{2}^{{{\text{tot}}}}$, the rate constant $R_{2}^{{{\text{tot}}}}$ and linewidth is given by ${\Delta ^{{\text{tot}}}}=R_{2}^{{{\text{tot}}}}/\pi =1/(\pi T_{2}^{{{\text{tot}}}})$ assuming Lorentzian lineshapes.

### Coherent contributions (Δ^coh^)

These are due to homonuclear interactions described by the spin-system Hamiltonian. They include isotropic and anisotropic chemical shifts as well as proton–proton dipolar couplings. Scalar couplings are neglected. During the detection of the FID there is no RF-field irradiation on the proton channel and we consider heteronuclei (in particular ^15^N and ^13^C) to be fully decoupled by RF pulses. Therefore, only a single time dependence, introduced by the sample rotation, is present in the rotating-frame system Hamiltonian and can be treated using average Hamilton theory (AHT) (Haeberlen and Waugh 1968; Maricq and Waugh 1979).

1 $${\bar {H}^{(1)}}=\int\limits_{0}^{1} {d{\tau _1}H({\tau _1})}$$

2 $${\bar {H}^{(2)}}=\frac{1}{{{\nu _{\text{r}}}}}\frac{{ - i}}{2}\int\limits_{0}^{1} {d{\tau _2}} \int\limits_{0}^{{{\tau _2}}} {d{\tau _1}[H({\tau _2}),{\text{ }}H({\tau _1})]}$$

3 $${\bar {H}^{(3)}}=\frac{1}{{{\nu _{\text{r}}}^{2}}}\frac{{ - 1}}{6}\int\limits_{0}^{1} {d{\tau _3}} \int\limits_{0}^{{{\tau _3}}} {d{\tau _2}} \int\limits_{0}^{{{\tau _2}}} {d{\tau _1}\left( {\left[ {H({\tau _3}),{\text{ [}}H({\tau _2}),{\text{ }}H({\tau _1}){\text{]}}} \right]+\left[ {[H({\tau _3}),{\text{ }}H({\tau _2})],{\text{ }}H({\tau _1})} \right]} \right)}$$

Here, the variables ${\tau_{i}}$ refer to normalized times ${\tau_{i}}$ = ${t_{i}} /{\tau_{r}}$ with the rotor period ${\tau_{r}}$. The lowest order term ${\bar {H}^{(1)}}$ is the desired Hamiltonian containing, for spin ½, only the isotropic chemical shift ${\Omega _i}$ with ${\bar {H}^{(1)}}=\sum\nolimits_{i} {{\Omega _i}{{\text{I}}_{i,z}}} .$ The higher-order terms contain cross-terms between different homonuclear dipolar terms and anisotropic and isotropic chemical shifts. In the limit of infinitely fast spinning, the higher order terms, which scale with the spinning frequency as $1/{\nu _{\text{r}}}$ and $1/\nu _{{\text{r}}}^{2}$, vanish, but for finite spinning speeds they still contribute. The lineshape resulting from ${\bar {H}^{(2)}}$ (and even more ${\bar {H}^{(3)}}$) is quite complex with spatial rank 4, 2 and 0 tensorial contributions. In large spin systems, however, it can often approximately be described by a Lorentzian line broadening with $\Delta _{{(2)}}^{{{\text{coh}}}}({\nu _{\text{r}}})={c^{(2)}}/{\nu _{\text{r}}}$ where the coefficient ${c^{(2)}}$ is proportional to the norm of the integral part of ${\bar {H}^{(2)}}$. The third-order contribution is, correspondingly, $\Delta _{{(3)}}^{{{\text{coh}}}}({\nu _{\text{r}}})={c^{(3)}}/\nu _{{\text{r}}}^{2}.$ The two terms decrease linearly and quadratically, respectively, with the inverse spinning frequency.

### Incoherent contributions (Δ^incoh^)

Proteins are dynamical entities, therefore, also incoherent contributions due to stochastic processes (Redfield-type *T*_2_ relaxation, chemical exchange) to the linewidth exist and constitute a more fundamental limit to resolution. The situation is similar to the one in solution-state NMR with two exceptions: (i) there is no overall tumbling motion; (ii) relaxation-rate constants are a function of the spinning frequency as predicted already early on (Haeberlen and Waugh 1969; Jasinski 1979). For faster spinning, these effects become prominent if there are motions with a correlation time comparable to the inverse rotor frequency (Kurbanov et al. 2011; Smith et al. 2016; Krushelnitsky et al. 2014). The influence of the spinning on the incoherent contribution to the linewidth derives from the property that the spectral density of the motion is no longer sampled at (angular) frequency $\omega =0$ but at the (angular) spinning frequency ${\omega _{\text{r}}}$ and twice this value.

For the simple example of a heteronuclear ^1^H–^15^N spin-pair, the heteronuclear dipolar contribution to $R_{2}^{{{\text{incoh}}}}$ is given, in the Redfield limit, by Haeberlen and Waugh (1969):

4 $$R_{2}^{{{\text{NH}}}}={\left( {\frac{{{\delta ^{\text{D}}}}}{4}} \right)^2}\left( {\frac{4}{3}J({\omega _{\text{r}}})+\frac{2}{3}J(2{\omega _{\text{r}}})+\frac{1}{2}J({\omega _I} - {\omega _s})+3J({\omega _S})+\frac{3}{2}J({\omega _I})+3J({\omega _I}+{\omega _S})} \right)$$

The corresponding contribution from a homonuclear ^1^H–^1^H spin-pair is

5 $$R_{2}^{{{\text{HH}}}}={\left( {\frac{{{\delta ^{\text{D}}}}}{4}} \right)^2}\left( {3J({\omega _{\text{r}}})+\frac{3}{2}J(2{\omega _{\text{r}}})+\frac{{15}}{2}J({\omega _I})+3J(2{\omega _I})} \right)$$

For relaxation under an on-resonance cw spinlock (rotating-frame relaxation) on the protons with a nutation frequency of ${\omega _1}$ we find:

6 $$R_{{1\rho }}^{{{\text{NH}}}}={\left( {\frac{{{\delta ^{\text{D}}}}}{4}} \right)^2}\left( {\frac{1}{3}J({\omega _1} - 2{\omega _{\text{r}}})+\frac{2}{3}J({\omega _1} - {\omega _{\text{r}}})+\frac{2}{3}J({\omega _1}+{\omega _{\text{r}}})+\frac{1}{3}J({\omega _1}+2{\omega _{\text{r}}})+\frac{1}{2}J({\omega _I} - {\omega _S})+3J({\omega _S})+\frac{3}{2}J({\omega _I})+3J({\omega _S}+{\omega _I})} \right)$$

and for the homonuclear contribution (Rovo and Linser 2017):

7 $$R_{{1\rho }}^{{{\text{HH}}}}={\left( {\frac{{{\delta ^{\text{D}}}}}{4}} \right)^2}\left( {\frac{3}{4}J(2{\omega _1} - 2{\omega _{\text{r}}})+\frac{3}{2}J(2{\omega _1} - {\omega _{\text{r}}})+\frac{3}{2}J(2{\omega _1}+{\omega _{\text{r}}})+\frac{3}{4}J(2{\omega _1}+2{\omega _{\text{r}}})+\frac{{15}}{2}J({\omega _I})+3J(2{\omega _I})} \right)$$

In these equations, ${\delta ^{\text{D}}}$ denotes the anisotropy of the dipolar-coupling tensor and ${\omega _I}$ and ${\omega _S}$ the two Larmor frequencies. The equations above are given for relaxation on the I spin, which in our case is the proton. For slow motions, relevant for line broadening, only the leading two or four terms in the equations above must be considered. In the limit of slow spinning, the sum of the first two terms yields $2J(0)$ and the expressions valid for static samples are recovered. The spectral-density function for a motion with a single correlation time $\tau$ and an amplitude *S* is defined as

8 $$J(\omega )=\frac{2}{5}(1 - {S^2})\frac{\tau }{{1+{{(\omega \tau )}^2}}}.$$

**Fig. 1:**
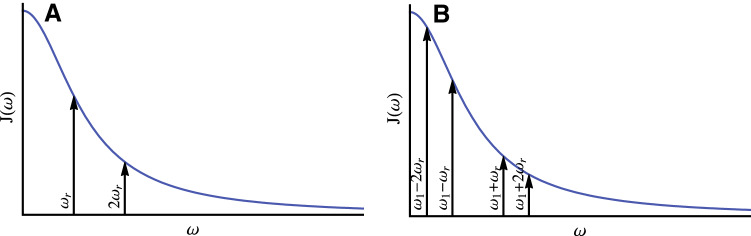
S-spin spectral densities sampled for R_2_ (**a**) and R_1ρ_ (**b**) under MAS for the simple case of a heteronuclear spin pair

The *T*_2_ relaxation times, in the presence of slow motion, become longer with increasing spinning frequency (Fig. 1) due to the fact that the spectral-density function is monotonously decaying $(J(\omega )<J(0))$ and the resonance lines become correspondingly narrower. The coherent and incoherent contributions form together the homogenous contributions: ${\Delta ^{{\text{homo}}}}={\Delta ^{{\text{coh}}}}+{\Delta ^{{\text{incoh}}}}$.

### Inhomogeneous contributions (Δ^inhomo^)

In addition to the contributions described above, additional effects arise from magnetic-field heterogeneity and heterogeneities specific to sample preparation. The former can be addressed by the design of the probe and optimizing the currents in the shim coils, while the latter one requires optimized sample-preparation techniques. Again, using the assumption that these effects approximately lead to a Lorentzian line broadening, the resulting ${\Delta ^{{\text{inhomo}}}}$ is described by a further rate constant $R_{2}^{{({\text{inhomo}})}}$. This decay is to first order refocused in Hahn echoes.

To first approximation, the linewidth of each resonance is, thus, given by the sum of the terms just discussed: ${\Delta ^{{\text{tot}}}}({\nu _{\text{r}}})={\Delta ^{{\text{coh}}}}({\nu _{\text{r}}})+{\Delta ^{{\text{incoh}}}}({\nu _{\text{r}}})+{\Delta ^{{\text{inhomo}}}}={\Delta ^{{\text{homo}}}}({\nu _{\text{r}}})+{\Delta ^{{\text{inhomo}}}}$.

It is the aim of this paper to experimentally characterize ${\Delta ^{{\text{tot}}}}({\nu _{\text{r}}})~$ between 93 and 126 kHz MAS and to measure or estimate its three components in order to describe the linewidth in proteins under fast MAS and to extrapolate to even faster MAS frequencies. ${\Delta ^{{\text{tot}}}}({\nu _{\text{r}}})~$ is obtained by measuring the proton linewidth in the spectrum, its homogeneous contribution ${\Delta ^{{\text{homo}}}}({\nu _{\text{r}}})$ by determining the relaxation-rate constant $R_{2}^{{{\text{homo}}}}$, typically called *R*_2_′, in a Hahn-echo experiment ${\Delta ^{{\text{homo}}}}({\nu _{\text{r}}})={R^{\prime}_2}/\pi$. Then ${\Delta ^{{\text{inhomo}}}}$ can be obtained as ${\Delta ^{{\text{tot}}}} - {\Delta ^{{\text{homo}}}}$. The separation of the two terms ${\Delta ^{{\text{coh}}}}({\nu _{\text{r}}})$ and ${\Delta ^{{\text{incoh}}}}({\nu _{\text{r}}})$ that make together the homogeneous contribution is more difficult since both terms are spinning-frequency dependent and both narrow in the fast spinning limit upon further increase of the spinning frequency. The MAS dependence of the incoherent contribution is determined by the sampling of the spectral-density function as indicated in Fig. 1a. It is only weak for motions with inverse correlation times much larger than the spinning frequency. Then, the incoherent contribution could be obtained from extrapolating ${\Delta ^{{\text{homo}}}}$ to very fast spinning. Alternatively, ${\Delta ^{{\text{incoh}}}}({\nu _{\text{r}}})$ can also be calculated, in an order-of-magnitude estimation from the correlation times and order parameters which are known for ubiquitin (Lakomek et al. 2017). The contribution $R_{2}^{{{\text{NH}}}}$ can be obtained from Eq. (4) and we assume $R_{2}^{{{\text{HH}}}}$ to be comparable. ${\Delta ^{{\text{coh}}}}({\nu _{\text{r}}})$ can then be calculated as ${\Delta ^{{\text{homo}}}}({\nu _{\text{r}}}) - {\Delta ^{{\text{incoh}}}}({\nu _{\text{r}}})$. It will be shown below that ${\Delta ^{{\text{coh}}}}({\nu _{\text{r}}}) \approx {\Delta ^{{\text{homo}}}}({\nu _{\text{r}}})$ for ubiquitin, and we thus conclude that the coherent linewidth, caused by imperfect averaging of the dipolar interaction (as well as cross terms with the anisotropic chemical shift) is the dominant contribution to the linewidth in this protein. Therefore, a further, at least linear, improvement of the linewidth with faster spinning can be expected.

## Results and discussion

**Fig. 2:**
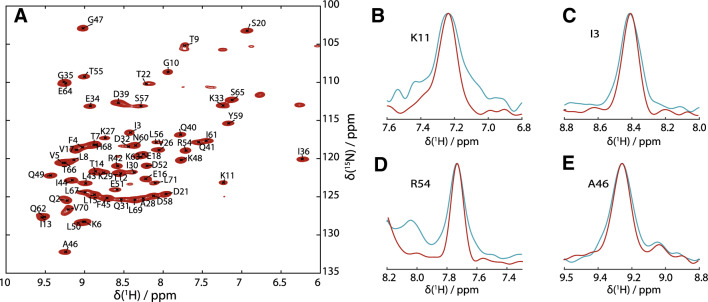
Proton-detected ^1^H–^15^N correlation spectrum of fully protonated ubiquitin acquired in 9 min at 126 kHz MAS at a sample temperature of 20 °C (**a**). **b**–**e** Selected traces through four isolated peaks along the ^1^H dimension to exemplarily show the linewidths of those peaks at 126 kHz MAS (red) compared to peaks from the spectrum at 93 kHz MAS (blue), acquired with the same sample and probe under the same experimental conditions (for full spectrum, see SI, Fig. S1), normalized to the intensity of each peak

**Fig. 3:**
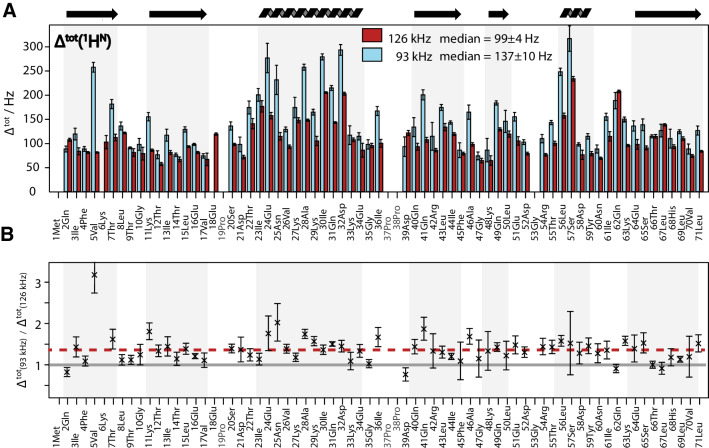
**a** Site-specific ^1^H^N^ linewidths in fully protonated ubiquitin at 93 (blue) and 126 kHz (red) as obtained from fitting the spectra in Fig. 2 using INFOS (Smith 2017). The median for Δ^tot^ is given in the figure. In addition, the mean values for Δ^tot^ are 154 ± 68 and 109 ± 38 Hz for 93 and 126 kHz MAS, respectively. In **b** the ratio of the linewidths at 93 kHz over the data at 126 kHz MAS is plotted with the error bars showing two standard deviations for each data point. The gray line marks the ratio 1 and the red line corresponds to the median value of 1.33 (calculated from the site-specific ratios)

Figure 2 shows the proton-detected (H)NH 2D correlation spectrum of fully protonated U[^13^C,^15^N] ubiquitin, measured at 126 kHz MAS and 1D traces along the proton dimension (red) which are narrower than the ones acquired at 93 kHz MAS (blue). A more detailed analysis of the experimental linewidth Δ^(tot)^ in the proton dimension for each residue has been obtained by fitting both 2D spectra by a sum of two-dimensional Lorentzian peaks using the program INFOS (Smith 2017) (see Supporting Information). This allows us to extract information not only from isolated peaks, but also for regions with limited spectral overlap. The quality of the fits is documented in SI, Fig. S2. In Fig. 3a the resulting residue-specific amide-proton linewidths are shown. As expected, almost all lines are narrower at 126 kHz MAS compared to 93 kHz. Figure 3b demonstrates this expected trend: Δ^tot^ narrows roughly by the expected factor of *r* = 126 kHz/93 kHz ≈ 1.35, namely the inverse of the ratio of the spinning frequencies, with a change in the median linewidth from 137 to 99 Hz (*r* = 1.38).

**Fig. 4:**
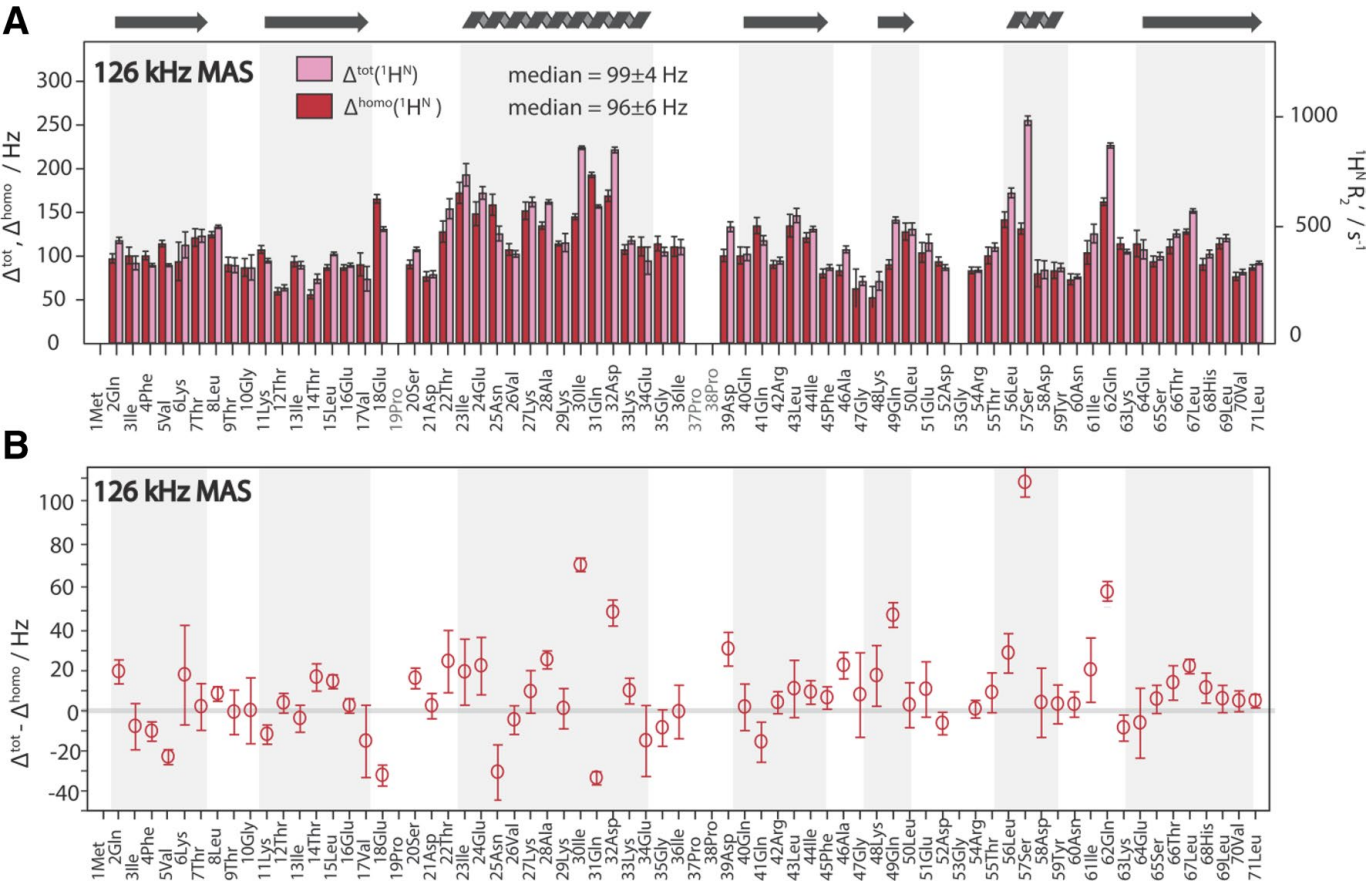
Site-specific ^1^H^N^ total linewidth Δ^tot^ as well as its homogeneous component Δ^homo^ in fully protonated ubiquitin at 126 kHz MAS (**a**) within a confidence interval of 95% as determined by a Monte-Carlo based error analysis (Sawilowsky and Fahoome 2003). Δ^homo^ was obtained from site-specific R_2_′ relaxation rate constants measured in a series of 2D experiments with different echo delay times where the coherence decay was subsequently fitted to a mono-exponential decay using FitTrace of INFOS (Smith 2017). In addition to the median given in the figure, the mean value of Δ^homo^ is 100 ± 28 Hz. The Δ^tot^ was obtained by fitting the 2D spectrum (processed without apodization or window function) using the fitting program INFOS (Smith 2017). In (**b**) the inhomogeneous contribution Δ^inhomo^ = Δ^tot^ − Δ^homo^ is shown. Secondary structural elements for ubiquitin are displayed at the top and are indicated by grey areas

The differences between the FWHM of the resonance line, ${\Delta ^{{\text{tot}}}}$, and its homogeneous contribution, ${\Delta ^{{\text{homo}}}}={R^{\prime}_2}/\pi$, were determined site-specifically by a series of 2D experiments at 126 kHz using a Hahn-echo approach (Hahn 1950; Cavanagh et al. 2006). The results are shown in Fig. 4a and show, that ${\Delta ^{{\text{tot}}}} \approx {\Delta ^{{\text{homo}}}}$ for the majority of residues as well as for the median. Therefore, the inhomogeneous contributions to the linewidth, ${\Delta ^{{\text{inhomo}}}}={\Delta ^{{\text{tot}}}} - {\Delta ^{{\text{homo}}}},$ are small in this sample with a median of 3 ± 7 Hz (Fig. 4b). Thus, the sample is well-ordered as expected for a crystalline protein. For most residues, the difference ${\Delta ^{{\text{inhomo}}}}$ can be explained by the shim alone (giving about 10 Hz—see SI, Fig. S3). Three residues have a ${\Delta ^{{\text{inhomo}}}}$ > 50 Hz (30Ile, 57Ser, 62Gln). The origin of the relatively large inhomogeneous broadening of these three residues must be of molecular origin (local structural disorder) but no obvious correlation with the crystal structure was found. Noticeable exceptions with a negative difference with 95% confidence are: 5Val, 18Glu, 25Asn, and 31Gln. The common feature of those peaks is either very low SNR (25Asn) or strong overlap, making the linewidth evaluation more prone to systematic errors which are not reflected in the statistical error bars. For this reason, data at 93 kHz, where the overlap is higher, have not been analyzed. We can conclude, however, that for most residues, there is only a negligible contribution of sample inhomogeneity to ${\Delta ^{{\text{tot}}}}$ for fully protonated-ubiquitin.

We can compare these results to data obtained with deuterated and 100% back-exchanged ubiquitin where the Δ^tot^ was measured as 42 ± 12 Hz at 93 kHz MAS (Agarwal et al. 2014). Note, that this value corresponds to the mean of 48 isolated peaks, whereas here, we report on the median value of all backbone amide peaks. In the deuterated and 100% back-exchanged ubiquitin, the average *T*_2_′(^1^H) relaxation was previously determined to 13.4 ± 0.3 ms (Penzel et al. 2015) and the homogeneous ^1^H linewidth Δ^homo^ = 24 ± 5 Hz. In this case, the inhomogeneous contribution is larger (18 ± 13 Hz) although the error bars overlap, which can be explained by the slightly worse shim (~ 7 Hz FWHM(^13^C) on adamantane, corresponding to 28 Hz on protons). For deuterated and 100% back-exchanged ubiquitin the site-specific homogeneous linewidth shows a strong correlation with the secondary structure of their respective residue which can be explained by the difference of proton density in α-helices and β-sheets (Penzel et al. 2015). For the fully protonated ubiquitin the differences in Δ^homo^ between α-helices and β-sheets are less pronounced, as the secondary-structure elements are no longer the determining factor for the proton density around a given amide proton.

**Fig. 5:**
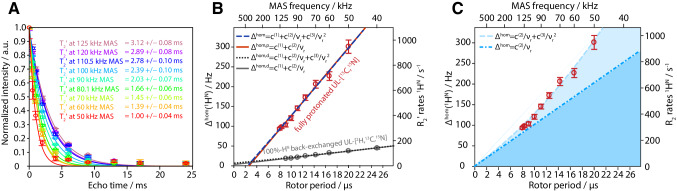
**a** Bulk ^1^H *T*_2_′ coherence decay curves as measured with a Hahn-echo experiment with increasing echo-delay times as a function of spinning frequency. Data points represent the average of three independent measurements and the error bars the standard deviation, solid lines the mono-exponential fit. **b** Corresponding coherent linewidth extracted from the fit of the data to the left for fully-protonated ubiquitin (red) with the error bars indicating the standard deviation of each fit according to the bootstrapping method (Efron 1979). The grey data set represents the *R*_2_′ relaxation rates for deuterated, and 100% back-exchanged ubiquitin as measured up to 120 kHz MAS using the same probe. The red and grey line represent the linear regression of the data points, respectively, and the blue and black dotted curves are from a three-parameter quadratic fit. **c** Shows the linear contribution (dotted, dark blue) to the quadratic fit through zero (dashed, light blue) for the data of fully protonated ubiquitin. See main text for the equations of the different fits

The relatively small variation of Δ^homo^ over the residues in the protonated compound allows us to characterize the dependence of the homogeneous contribution to the linewidth on the MAS frequency on a finer grid by looking at one-dimensional bulk ^1^H^N^ *R*_2_′ relaxation rate constants as determined using a Hahn-echo experiment, ensuring that the temperature is kept constant for different MAS frequencies. Figure 5a shows that with increasing MAS frequency the bulk coherence decay time of the amide protons in fully protonated ubiquitin increases monotonously between 40 and 125 kHz. At the highest MAS frequency (125 kHz) the *T*_2_′ of fully protonated ubiquitin is 3.12 ms. The corresponding Δ^homo^ is equal to 102 Hz, very close to the median value of 97 Hz over all residues at the same spinning frequency.

The homogeneous linewidth in Fig. 5b thus decreases with increasing MAS frequency. As discussed above, the spinning-frequency dependence of the experimental homogeneous linewidth Δ^homo^ is expected to have linear and quadratic terms. We first fitted the experimental results with a linear term (plus a constant term representing the incoherent contribution), ${\Delta ^{{\text{homo}}}}={c^{(1)}}+{c^{(2)}}/{\nu _{\text{r}}}$ with the $~{c^{(n)}}$ being the free fit parameters and *n* being the order of the term in average Hamiltonian theory. Phenomenologically, this function leads to an good fit (*R*^2^ = 0.9785) of the data points for fully protonated ubiquitin with *c*^(1)^ = (− 45 ± 8) Hz and *c*^(2)^ = (17 ± 1) kHz^2^. Using a quadratic term in the fit, ${\Delta ^{{\text{homo}}}}={c^{(1)}}+{c^{(2)}}/{\nu _{\text{r}}}+{c^{(3)}}/{\nu _{\text{r}}}^{2}$, does not lead to a significantly better fit (*R*^2^ = 0.9786) (c^(1)^ = (− 41 ± 12) Hz, c^(2)^ = (17 ± 6) kHz^2^ and c^(3)^ = (0.02 ± 0.02) kHz^3^).

For deuterated and 100% backprotonated ubiquitin, the linear fit leads to c^(1)^ = (3 ± 1) Hz and c^(2)^ = (1.7 ± 0.1) kHz. While for the deuterated protein c^(1)^ is compatible with Δ^incoh^ = 1 ± 2 Hz, (mean value) estimated from ^15^N relaxation data (Lakomek et al. 2017) (see SI, Fig. S4 for the calculated proton *R*_2_′ relaxation rates from the site-specific correlations times and order parameters), the negative value obtained for fully protonated sample is unphysical. We therefore repeated the quadratic fit with c^(1)^ = 0 and obtained c^(2)^ = 10 ± 1 kHz^2^ and c^(3)^ = (0.25 ± 0.05) Hz^3^. The fit is still good (*R*^2^ = 0.9774) and the linewidth is dominated by the linear term (dark blue region in Fig. 5c) with a smaller but still significant contribution from a quadratic term (light blue). This can be compared with results on *β*-L-Asp-L-Ala dipeptide between 30 and 100 kHz (Sternberg et al. 2018), showing similar behavior with a stronger quadratic contribution. This quadratic dependence cannot yet be reliably fit with the spinning frequencies that are available and results in the unphysical incoherent contribution when linearly extrapolating to infinite MAS frequencies.

**Fig. 6:**
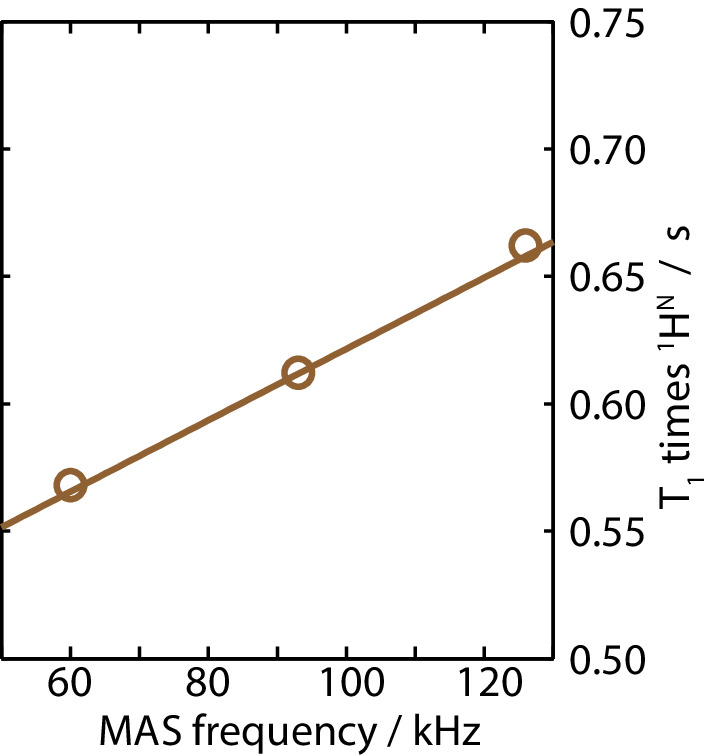
Bulk ^1^H^N^ *T*_1_ relaxation times at different MAS frequencies, fitted to a mono-exponential function

Next, we investigate the longitudinal *T*_1_ relaxation behavior. As described earlier for glycine (Ye et al. 2014), the relaxation time increases for some protons with faster MAS due to a slowing down of the proton spin diffusion that connects the relaxation sinks (e.g., methyl groups) to the remainder of the protons. Longer T_1_ relaxation times increase the experimental time or lead to less sensitivity per time unit. The MAS-dependent bulk *T*_1_(^1^H^N^) relaxation times show an increase of 1.4 ms per kHz MAS frequency (slope of fit in Fig. 6), and the resulting increase of a factor of 1.08 between 93 and 126 kHz is not problematic. The correspondent loss in SNR per time unit is more than compensated by the gain in signal-to-noise due to the increase in signal intensity as the ^1^H linewidth decreases by a factor of 1.38.

**Fig. 7:**
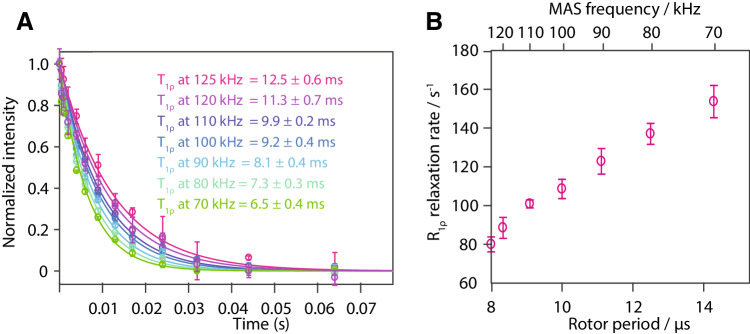
MAS-dependent bulk proton relaxation rate constants under a spinlock (*T*_1ρ_). **a** shows the decay curves under a spinlock for the amide protons in fully protonated ubiquitin for each spinning frequency (circles) as well as the mono-exponential fit to extract relaxation rate constants (lines). Each data point was recorded in three independent experiments to obtain the error bars. In **b** the fitted *R*_1ρ_ relaxation rate constants are plotted against the length of the rotor period

True incoherent effects to the linewidth Δ^incoh^ can be described by Eqs. 4–7. However, for protons it is not straightforward to unravel different contributions to relaxation, especially in a strongly-coupled network of protons as is the case in fully protonated samples. There are some cases where proton *R*_1*ρ*_ relaxation has been used in solution-state NMR to investigate dynamics (Eichmüller and Skrynnikov 2005; Ishima et al. 1998), and ^1^H *R*_1ρ_ relaxation rates (under a Lee-Goldberg spin lock) have also been utilized to extract dynamical information in solid-state NMR, as early as 2001 (Huster et al. 2001). Also, recently there has been an attempt to analyze *R*_1*ρ*_ of amide protons in a highly deuterated environment, in the context of a dynamics analysis (Rovo and Linser 2017). However, even in this diluted environment the relaxation mechanism for protons is difficult to disentangle (Gauto et al. 2017). Fully protonated ubiquitin with its dense ^1^H–^1^H network is even more complex and a quantitative motional analysis is not possible. Still, the phenomenological *R*_1*ρ*_ relaxation-rate constants of the amide protons are of considerable interest for pulse-sequence design (e.g. to predict the magnetization loss during spinlock periods, for example, for a CP or DREAM transfer). The MAS-dependent bulk proton *R*_1ρ_ relaxation rate constants of Fig. 7 show a clear decrease with faster spinning frequency. This is expected because the spectral density entering the calculation of *R*_1ρ_ is sampled at higher frequencies assuming that ${\omega _1} \ll {\omega _{\text{r}}}$ (see Fig. 1).

**Fig. 8:**
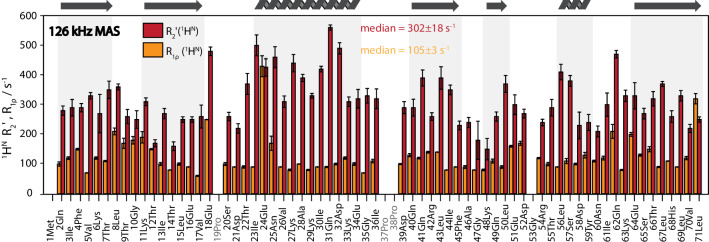
Site-specific ^1^H^N^ *R*_2_′ and *R*_1ρ_ rate at 126 kHz MAS of fully protonated ubiquitin at 20 °C sample temperature. The data set was acquired as a series of 2Ds with increasing relaxation delays (see SI for details) and fit mono-exponentially using the FitTrace function of INFOS (Smith 2017). Error bars indicate a confidence interval of 95% and are calculated using a Monte-Carlo approach (Sawilowsky and Fahoome 2003). The spinlock field strength was set to 12 kHz in all experiments

The *R*_1ρ_ have also been measured site specifically and are given, together with the ^1^H^N^ *R*_2_′ (as already presented in Fig. 4) in Fig. 8. Generally, the rate constants *R*_1ρ_ are lower than the *R*_2_′ at 126 kHz MAS (see also SI, Fig. S4 for the ratio of the two rates). There are two exceptions, where *R*_2_′ is slightly lower than *R*_1ρ_. Both residues (Glu24, Leu71) are found in dynamic regions of ubiquitin (Massi et al. 2005) and also show increased *R*_*1ρ*_ relaxation-rate constants measured on ^15^N in solid-state NMR (Lakomek et al. 2017). Glu24, located in the N-terminus of the α-helix, is often broadened, sometimes beyond detection, in both solution and solid-state NMR. This is mostly likely due to chemical-exchange broadening which has been characterized for the neighboring residues Ile23 and Asn25 (Massi et al. 2005) and occurs with a rate constant of about 25,000 s^− 1^ comparable to the MAS frequency. Leu71 also undergoes chemical-exchange broadening at a similar rate (Massi et al. 2005) and further marks the beginning of the often unobservable C-terminus when using solid-state NMR (Schanda et al. 2010; Igumenova et al. 2004; Schubert et al. 2006).

We conclude, therefore, that, while a quantitative interpretation of the proton *R*_1ρ_ relaxation-rate constants in terms of molecular dynamics of fully protonated ubiquitin remains elusive, there are some interesting features visible in the proton *R*_1ρ_ data that are correlated to true stochastic motions.

## Conclusions

The benefits of MAS at frequencies beyond 100 kHz and up to 126 kHz, are assessed for the microcrystalline model protein ubiquitin in its fully protonated form. At 126 kHz MAS the ubiquitin spectrum shows a median proton linewidth Δ^tot^ of 99 Hz, compared to 137 Hz at 93 kHz MAS. These values are, for ubiquitin, dominated by homogeneous broadening effects, that decrease with increasing spinning frequency. Sample inhomogeneities and imperfections of the shim do not significantly contribute to the lineshape.

The observed total linewidth Δ^tot^ as well as the homogeneous contribution Δ^homo^ decrease monotonously with the spinning frequency over the entire range from 50 to 125 kHz. The decrease in Δ^homo^ is dominated by a linear term, corresponding to the second order term in average Hamiltonian theory, and it is clear that still higher spinning frequencies will lead to further line narrowing. A significant contribution of a quadratic term at frequencies lower than 100 kHz exists, which hints at the contribution of a third-order AHT term.

The ^1^H linewidth in a solution-state NMR sample of ubiquitin is reported to be in the range of 6–9 Hz for amide protons (Cavanagh et al. 2006). In order to reach 10 Hz by solid-state NMR, we extrapolate that one would have to spin roughly 300 and over 1000 kHz, for deuterated/back-exchanged and protonated ubiquitin, respectively. However, the comparison will be much more favorable for larger proteins where the solid-state linewidth is expected to be still the same, whereas the lower rotational correlation time of the overall tumbling leads to increased linewidths in solution. Similar predictions have been reported by simulations for selectively methyl-labeled proteins (Xue et al. 2018).

Motional processes (incoherent contributions) contribute only weakly to the linewidth for this microcrystalline model protein. There are, however, indications that the situation is different for membrane proteins, which have a similar proton density as ubiquitin but lead to broader resonance lines, even for deuterated systems (Barbet-Massin et al. 2014; Fogeron et al. 2016; David et al. 2018). It is, therefore, likely that these proteins show a larger contribution from motional processes, through ${\Delta ^{{\text{incoh}}}}$ to ${\Delta ^{{\text{homo}}}}$ and to *T*_2_′. Also, the small inhomogeneous contribution in this ubiquitin sample is a favourable case, and it should be stressed that the total linewidth of other proteins (even microcrystalline, but also biological solid samples) might be significantly larger.

The beneficial influence of even faster MAS will be key for a new type of solid-state NMR spectroscopy in structural biology with sensitive detection of sample volumes of typically 100 µg only. Indeed, further line narrowing due to a decrease of ${\Delta ^{{\text{coh}}}}$, but also ${\Delta ^{{\text{incoh}}}}$ with spinning frequency is key to the success of faster-spinning application because the signal loss caused by the smaller sample volume inherent in faster MAS rotors can then be largely compensated by the decreased linewidth.

## Materials and methods

### Sample

Uniformly-labeled [^13^C,^15^N] ubiquitin was prepared by over-expression in *E. coli* and crystallized in MPD as previously described (Igumenova et al. 2004). The rotor was filled by ultra-centrifugation using filling tools (Bockmann et al. 2009) adapted for 0.6 mm rotors with the approximate amount of 0.28 mg protein.

### NMR spectroscopy

All NMR experiments were carried out on a Bruker Avance III 850 MHz (^1^H Larmor frequency) spectrometer. Data at all spinning frequencies was acquired with a 130 kHz, 0.6 mm (0.4 µl sample volume) rotor, proton-detection optimized triple-resonance MAS probe (http://www.nmri.eu) using nitrogen for the bearing to cool.

The sample temperature of 20 °C was monitored by the frequency of the supernatant water resonance line (Bockmann et al. 2009). To achieve this sample temperature the nitrogen gas for the bearing pressure was piped through a BCU II (Bruker), cooled to − 80 °C and led via a glass dewar into the stator. At a bearing pressure of 2.8 bar (and a drive pressure of 3.6 bar), necessary for 126 kHz MAS, this temperature was easily reached and could be maintained with a heater power of 0.8%. MPD was used as internal chemical-shift standard. The magic angle was set following the “on-sample” method described in Penzel et al. (2018) using the first negative maximum to optimize.

All proton-detected 2D HNH experiments for site-specific analysis at 93 and 126 kHz MAS employ two dummy scans and a recycle delay of *T*_1_(^1^H) * 1.26 which lead to 0.75 and 0.85 s for data at 93 and 126 kHz MAS, respectively. Furthermore, all 2D experiments use 120 ms MISSISSIPPI solvent suppression (Zhou and Rienstra 1997) at 12 kHz field strength, 5 kHz WALTZ64 (Zhou et al. 2007; Shaka et al. 1983) decoupling on ^15^N during acquisition, and 10 kHz frequency-swept low-power (fslp) TPPM (Thakur et al. 2006) decoupling on ^1^H during the indirect evolution periods. Fslp-TPPM has shown the best performance at fast MAS (Penzel et al. 2015). Acquisition times were 70 and 25 ms with a spectral width of 47 and 40 ppm (5550 and 172 points) in the direct and indirect dimension, respectively, while States-TPPI was employed for phase-sensitive detection. With 32 scans each 2D experiment took about 3 h.

Two adiabatic cross-polarization (CP) steps were chosen to transfer polarization between ^1^H and ^15^N and back which were matched to fulfill the double-quantum (DQ) (*n* = 1) Hartmann-Hahn condition using 22 kHz RF-field on the ^15^N channel. Hard pulses were set to 150 and 41.667 kHz for the ^1^H and ^15^N nutation frequencies, respectively. For the series of six 2D experiments to determine the site-specific coherence decay time, a Hahn-echo was implemented after the first ^1^H π/2 pulse with the following delay times: 2 µs, 0.5 ms, 1.25 ms, 2.4 ms, 3.6 ms, 4.8 ms. For the site-specific T_1ρ_ relaxation times the Hahn-echo was substituted with a spinlock on the ^1^H channel using 12 kHz RF-field strength and durations of 2 µs, 1.28 ms, 3.6 ms, 6 ms, 9.5 ms, 15 ms.

For the determination of the bulk *T*_2_′ relaxation times, the same experiment as described above was used in a 1D fashion at spinning frequencies from 50 to 125 kHz, where the ^1^H spinlock field strength was optimized for each MAS while the other parameters were left as optimized for 126 kHz MAS. The bulk *T*_1_ relaxation at 60, 93, and 126 kHz MAS using a saturation-recovery building block instead of the Hahn-echo with delay times of 100 µs, 50 ms, 100 ms, 350 ms, 1 s, 2 s, 4 s, 6 s. Each of these 1D experiments was repeated for at least three times independently yielding the estimates for the statistical errors.

### Data processing and analysis

Initial processing (Fourier transformation and apodization using QSINE 2.5 in both dimensions) was done in TopSpin (3.5pl2, Bruker) while the analysis was performed in MATLAB (MATLAB 2016R, The MathWorks Inc., Natick, MA 2016) using INFOs spectrum fitting (Smith 2017) to extract site-specific information out of the 2D experiments. For this, the 2D spectra were fitted using SpecFit and the coherence decay of the series of 2Ds was analyzed using FitTrace. The standard deviation for the linewidths and *R*_2_′ relaxation times was calculated by synthesizing 100 spectra for each spectrum and calculating the rmsd for each parameter. The pseudo-2D traces for bulk relaxation parameters were fitted to a mono-exponential decay using bootstrapping (Efron 1979).

## Electronic supplementary material

Below is the link to the electronic supplementary material.

Supplementary material 1 (PDF 1165 KB)
