# Supplementary material for: Spinning faster: protein NMR at MAS frequencies up to 126 kHz
Source: J Biomol NMR. 2019 Jan 24;73(1):19–29. doi: 10.1007/s10858-018-0219-9 (PMC6441448; doi:10.1007/s10858-018-0219-9)
Supplement: Supplementary file 1 — Supplementary material 1 (PDF 1165 KB) [file 10858_2018_219_MOESM1_ESM.pdf]

Supporting Information

Spinning faster: Protein NMR at MAS frequencies up to 126 kHz

Susanne Penzel[1], Andres Oss[2], Mai-Liis Org[2], Ago Samoson[2*], Anja Böckmann[3*], Matthias Ernst[1*] and Beat H. Meier[1*]

[1]*Physical Chemistry, ETH Zürich, Vladimir-Prelog-Weg 2, 8093 Zurich, Switzerland*

[2]*NMR Instituut, Tartu Teaduspark, Tehnomeedikum, Tallinn University of Technology, Akadeemia tee 15a, 19086, Tallinn, Estonia*

[3]*Institut de Biologie et Chimie des Protéines, UMR 5086 CNRS/Université de Lyon 1, Labex ECOFECT, 7, passage du Vercors, 69367 Lyon, France*

1. Comparison of the complete 2D ^{1}H-^{15}N spectra at 93 and 126 kHz MAS

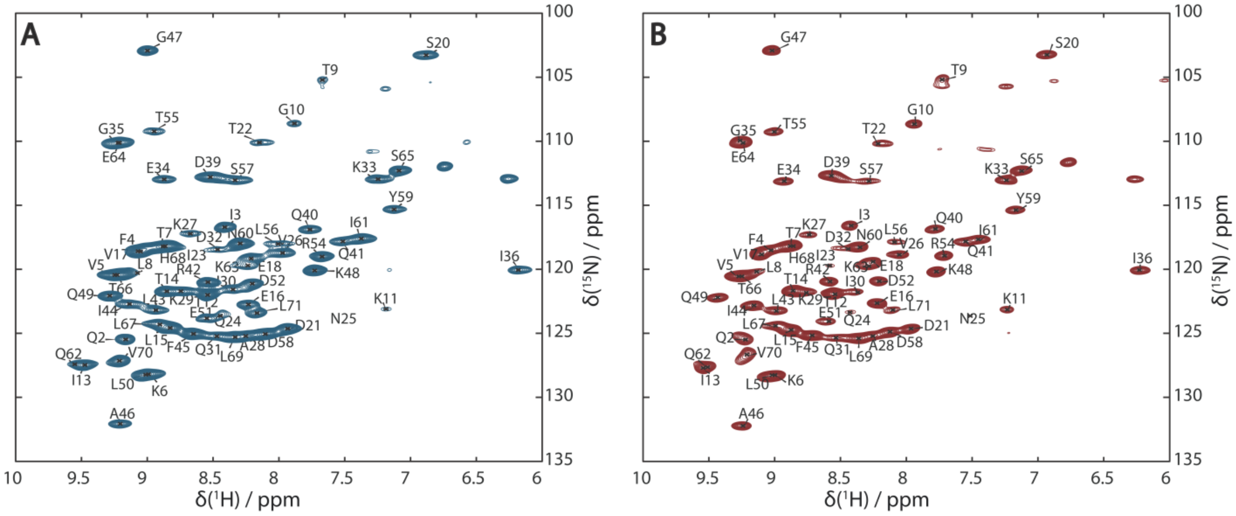

Figure S 1: Assigned experimental proton-detected ^{1}H-^{15}N correlation spectra using dipolar-coupling based transfers at 93 (A) and 126 (B) kHz MAS and a sample temperature of 20 °C. Both spectra were acquired using the same experimental conditions and are processed with an apodization function of QSINE 2.5. They are displayed at the same contour level with a 20% cutoff.

2. Spectrum fitting using INFOS

For the spectrum fitting only the assigned backbone amides were considered. Side-chain peaks were found to be isolated and can thus be left out of the fit without distorting any results. A peak list from an assignment in CCPN, based on the chemical shifts of deuterated and 100% back-exchanged ubiquitin and taking into account the isotope effect[54],as well as an hCANH 3D experiment on fully protonated ubiquitin, were used for starting values. They provided the number and initial positions of the peaks which were then allowed to move freely within a range of 0.3 ppm in each dimension during the fitting process. After the fit was completed and the best peak positions found, the assignment was again checked and corrected manually for some overlapping peaks. For both spinning frequencies, this initial fit was performed on a reference experiment and then used as input with fixed peak positions for the series of 2D spectra to extract relaxation rate constants using FitTrace. Note that for such a procedure, proper referencing of the spectra as well as a stable temperature is vital. In order to extract errors for the fit results from FitTrace, a Monte Carlo-based approach was used where 100 spectra were synthesized and the RMSD for each parameter was calculated.

3. Quality of the fits

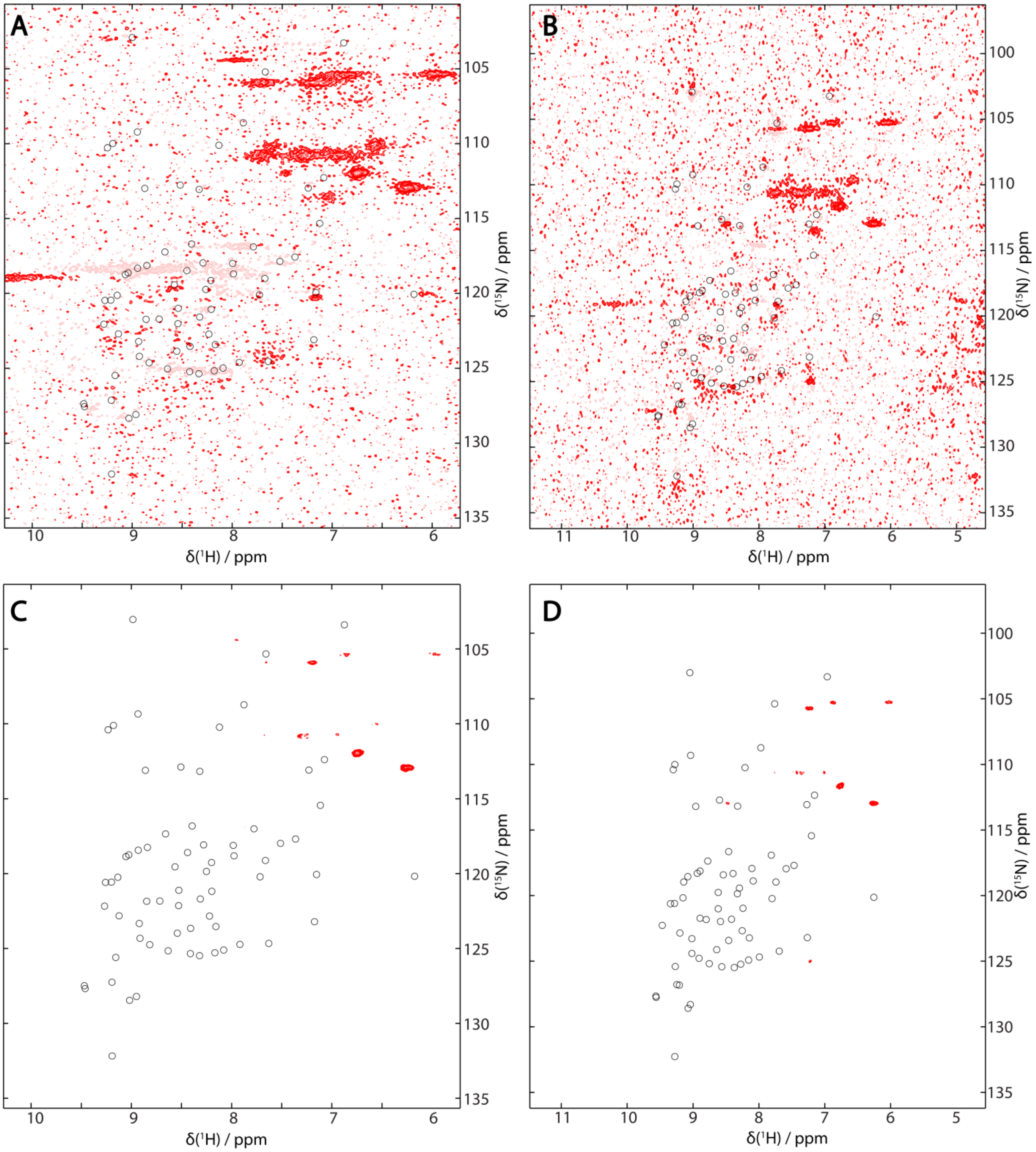

Figure S 2: Residuals of the calculated spectrum (fit) by INFOS for the spectrum at 93 (A and C) and 126 (B and D) kHz MAS. Contour levels are set to a cutoff of 5% (A and B) and to the same cutoff of 20% as in SI Figure S 1(C and D). Resulting peak positions from the fit are marked by open circles. Note that the sidechain amides were not fit and thus display a large residual.

4. Adamantane ^{13}C spectrum after shimming the 0.6 mm probe

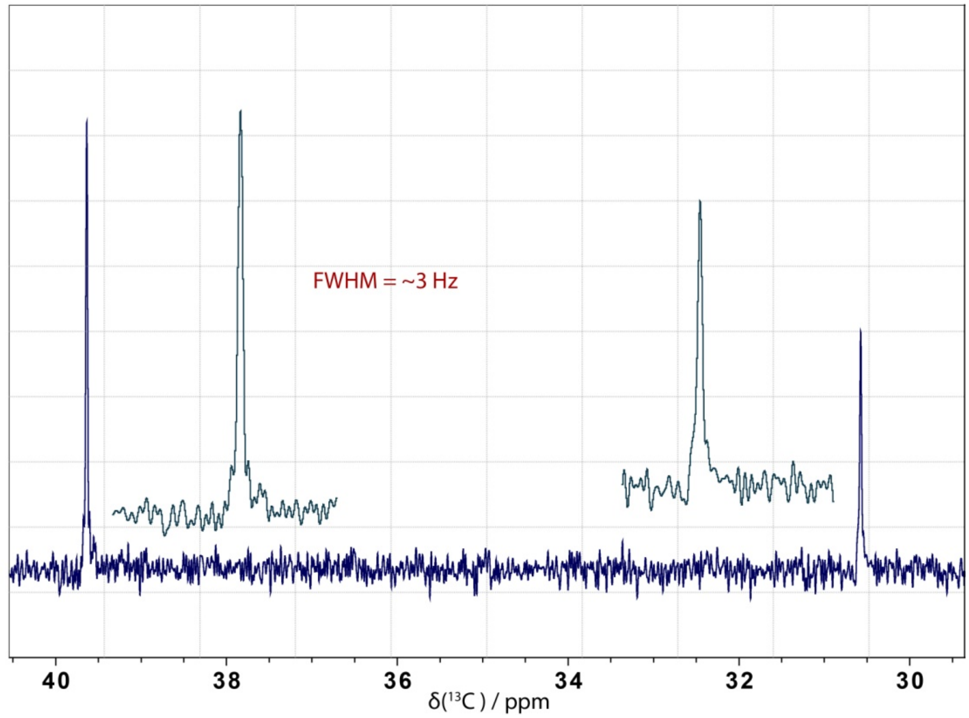

Figure S 3: ^{13}C-detected adamantane spectrum at 100 kHz MAS

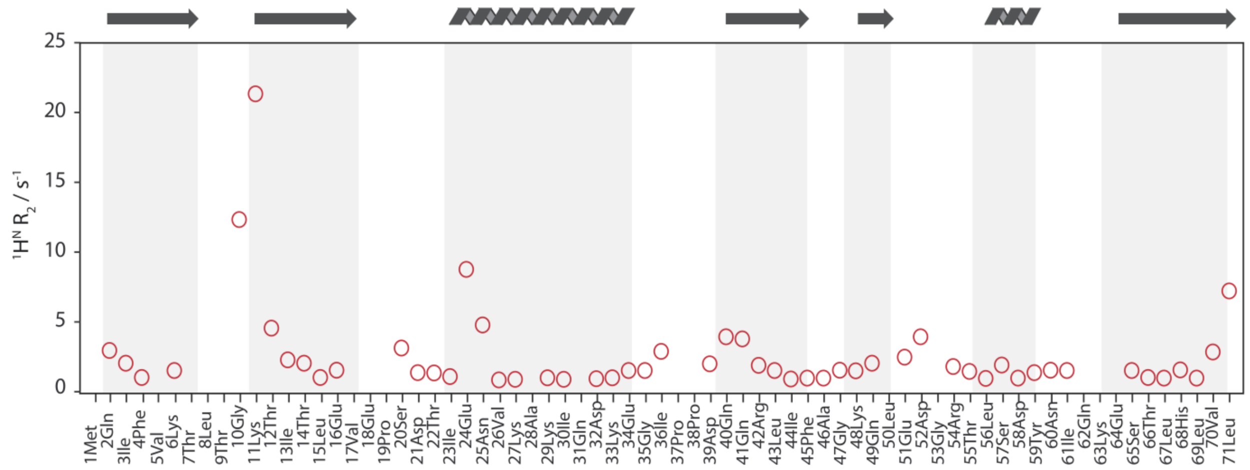

Figure S 4: Proton R_2' relaxation rates calculated from the site-specific correlation times and order parameters of ^{15}N-based relaxation data reported in Lakomek, *et al.* [29] taking into account dipolar interactions of the ^1H -^{15}N spin pair at 130 kHz MAS.

5. Ratio of proton $R_{1\rho}$ and R_2' relaxation rates

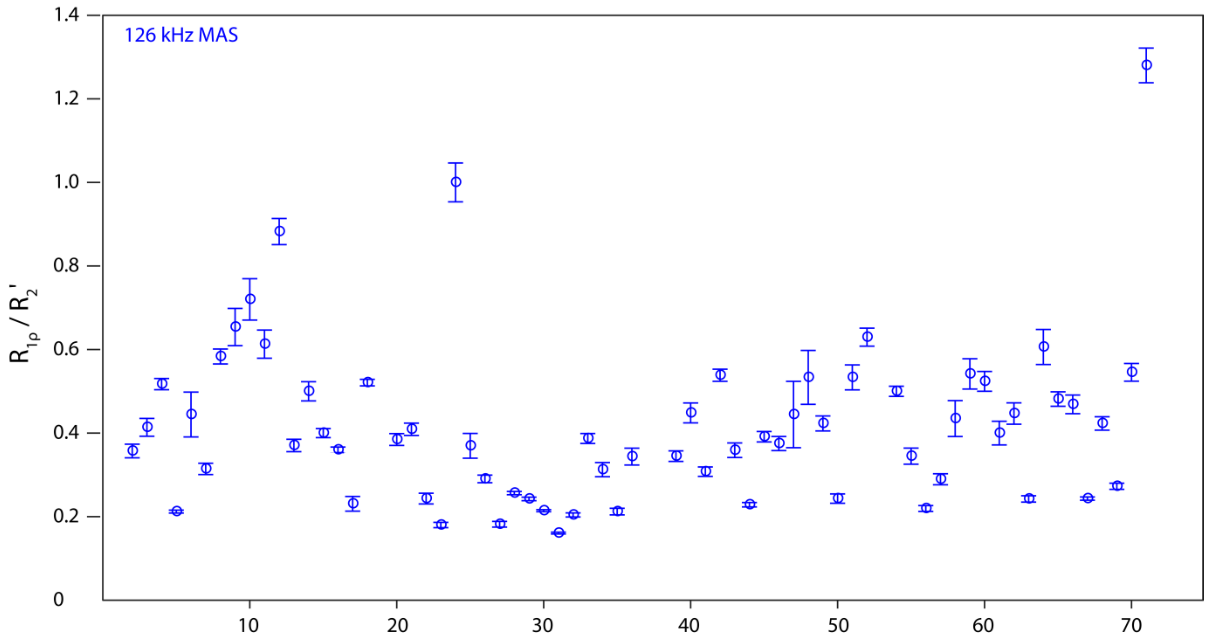

Figure S 5: Ratio of the R_2' and $R_{1\rho}$ relaxation rates for 126 (blue circles)
